# Risk Assessment for Children Exposed to Arsenic on Baseball Fields with Contaminated Fill Material

**DOI:** 10.3390/ijerph15010067

**Published:** 2018-01-04

**Authors:** Alesia C. Ferguson, Jennifer C. Black, Isaac B. Sims, Jennifer N. Welday, Samir M. Elmir, Kendra F. Goff, J. Mark Higginbotham, Helena M. Solo-Gabriele

**Affiliations:** 1 Environmental and Occupational Health, University of Arkansas for Medical Sciences, Little Rock, AR 72205, USA; AFerguson@uams.edu (A.C.F.); isims2@cub.uca.edu (I.B.S.); 2 Department of Civil, Architectural, and Environmental Engineering, University of Miami, Coral Gables, FL 33146, USA; j.black1@miami.edu (J.C.B.); j.welday@umiami.edu (J.N.W.); 3 Florida Department of Health at Miami-Dade County, Miami, FL 33056, USA; Samir.Elmir@flhealth.gov; 4 Florida Department of Health, Tallahassee, FL 32399, USA; Kendra.Goff@flhealth.gov (K.F.G.); Joseph.Higginbotham@flhealth.gov (J.M.H.)

**Keywords:** cancer, non-cancer, arsenic, risk assessment, baseball fields

## Abstract

Children can be exposed to arsenic through play areas which may have contaminated fill material from historic land use. The objective of the current study was to evaluate the risk to children who play and/or spend time at baseball fields with soils shown to have arsenic above background levels. Arsenic in soils at the study sites located in Miami, FL, USA showed distinct distributions between infield, outfield, and areas adjacent to the fields. Using best estimates of exposure factors for children baseball scenarios, results show that non-cancer risks depend most heavily upon the age of the person and the arsenic exposure level. For extreme exposure scenarios evaluated in this study, children from 1 to 2 years were at highest risk for non-cancer effects (Hazard Quotient, HQ > 2.4), and risks were higher for children exhibiting pica (HQ > 9.7) which shows the importance of testing fill for land use where children may play. At the study sites, concentration levels of arsenic resulted in a range of computed cancer risks that differed by a factor of 10. In these sites, the child’s play position also affected risk. Outfield players, with a lifetime exposure to these arsenic levels, could have 10 times more increased chance of experiencing cancers associated with arsenic (i.e., lung, bladder, skin) in comparison to infielders. The distinct concentration distributions observed between these portions of the baseball fields emphasize the need to delineate contaminated areas in public property where citizens may spend more free time. This study also showed a need for more tools to improve the risk estimates for child play activities. For instance, more refined measurements of exposure factors for intake (e.g., inhalation rates under rigorous play activities, hand to mouth rates), exposure frequency (i.e., time spent in various activities) and other exposure factors (e.g., soil particulate emission rates at baseball play fields) can help pinpoint risk on baseball fields where arsenic levels may be a concern.

## 1. Introduction

Arsenic is an environmental toxicant of concern globally [1,2,3], and ranks #1 on the U.S. substance priority list due to frequency at facilities on the national priority list, toxicity, and potential for human exposures [4,5]. Arsenic is a metalloid, occurring naturally and anthropogenically in both inorganic (as arsenite and arsenate) and organic forms (as MMA—monomethylarsonic acid and DMA-dimethylarsinic acid). Arsenic is found naturally in the environment, in soils and rocks which in turn can be dispersed in nature through weathering, runoff, mining, and volcanic activity [6]. Historically, arsenic’s main anthropogenic use has been as a pesticide. Arsenic protects wood against termites, as an effective component of the chemical wood treatment preservative called chromated copper arsenate (CCA). CCA-treated wood was commonly found in fencing and play structures [7,8,9]. Effective January 2004, CCA-treated wood was no longer manufactured in the U.S. for residential uses, but its use to treat industrial wood products continues today. Arsenic has also been used historically as a pesticide for the control of insects (insecticides), as crop desiccants, and to control weeds (herbicides) [10,11,12]. Arsenic-containing insecticides include inorganic forms of lead arsenate, calcium arsenate, and copper acetoarsenite. Its use for insect control in orchards, on fruits, tobacco, and some vegetables has diminished greatly since the 1970s. The desiccant properties of arsenic are particularly effective for harvesting cotton due to its ability to deplete plants of leaves and moisture. Arsenic compounds used as herbicides include both the inorganic arsenicals and the organic arsenicals. Inorganic arsenical herbicides were used in the U.S. through 1993 and include the use of sodium arsenite to remove vegetation from rights-of-way and drainage ditch banks [11]. Until its phase out in 2009, organic arsenical herbicides, including monosodium methyl arsenate (MSMA), were used on lawns and turf—in particular golf courses [13].

Arsenic has both carcinogenic and non-carcinogenic risks. For non-carcinogenic risks, humans experience chronic and acute symptoms when exposed to arsenic in the environment. The most common adverse health effects associated with long-term exposure to inorganic arsenic is a pattern of skin changes. These include patches of lightened or darkened skin and the appearance of small “corns” or “warts” on the palms, soles, and torso, and are often associated with changes in the blood vessels of the skin [6]. Acute exposures can result in increased risk for cardiovascular related deaths in association with drinking water arsenic concentrations ranging from 50 to 900 µg/L [14]. The U.S. Department of Health and Human Services, the International Agency for Research on Cancer (IARC), and the U.S. EPA have concluded that inorganic arsenic is a known human carcinogen [6]. Chronic arsenic exposures have been linked to cancers of the lung, skin (basal and squamous cell), liver, and urinary tract (bladder, kidney, prostate, ureter, and urethra) [6]. In an ecological study, arsenic alone was associated with 5297 excess cases of lung cancer in the United States per year, where higher rates of lung cancer were seen in areas of higher arsenic concentrations even after controlling for smoking and income [15].

Environmental contamination of arsenic is a concern, particularly for children, whose play activities (e.g., increased hand to mouth contact, rolling and sliding on the ground) may bring them into close contact with contaminated soils, increasing exposure and uptake [16]. The consumption of fruits, vegetables and rice that may be contaminated, can also be higher than adults in some cases, where some variation is seen across ethnic groups [17,18]. Their lower bodyweights compared to intake rates also result in increased dosing rates compared to adults [19]. In addition, children are susceptible due to developing organs where rates of metabolism or elimination may be diminished compared to adults [6].

More recent epidemiological studies have shown increased risks for children to arsenic in water for outcomes such as respiratory disease following in utero early life exposures, diarrhea, diabetes, increased risks of infections, and adverse developmental outcomes [14]. For example in a systematic review of studies that met the criteria for conducting an analytical epidemiological study (i.e., biological monitoring in relation to disease outcome), findings consistently linked higher arsenic levels to higher rates of respiratory infections and diarrhea in children [20]. In a study conducted in Bangladesh for 200 children, strong associations were also found between higher levels of arsenic in well water and urine and lower intellectual function [21]. Exposure to heavy metals, such as arsenic, has also been implicated in the development of autism spectrum disorder in a study of 364 children [22]; however more confirmative studies are needed to look at mechanisms of action. Oxidative stress mechanisms, requiring further research, are believed to be one manner in which arsenic causes damage [20]. Genetic polymorphisms may also contribute to how both adults and children biologically respond to arsenic exposures [4]. Enough evidence exists and continues to emerge warranting the reduction of exposures to arsenic in communities, where possible for both children and adults.

Human exposure to arsenic can occur through many routes including water, food, and air, using cigarettes, or working in industrial settings [23,24]. In the context of the baseball fields, exposures can occur through contaminated soils where absorption from these soils occurs through the dermal, ingestion and inhalation routes. To screen soils for potential risks soil cleanup target levels (SCTL) have been established in the United States and abroad. SCTLs are dependent on site-specific uses, characteristics, background contributions, and toxicological profiles. The USEPA regional screening level today is 0.7 mg/kg [25]. This screening level is based on the carcinogenic risk level of 1 × 10^−6^ using some acceptable assumption of risk and exposure. The State of Florida has established SCTLs of 2.1 mg/kg for residential areas and 12 mg/kg for commercial/industrial areas [26].

Globally, background concentrations of arsenic in soils are around 5 mg/kg with ranges from 1 to 40 mg/kg [27]. In Florida, the average concentration measured in surface soils was 1.3 mg/kg [28]. A study by Chirenje et al. [29] found that levels were lower in non-urban soils (0.27 mg/kg) relative to urban soils. Specifically for Miami, this same study found that background levels in urban areas of Miami were at 4 mg/kg (arithmetic mean) and 2.8 mg/kg (geometric mean). A study conducted by Miami-Dade County to evaluate regional anthropogenic background levels in urban areas split the county into two regions [30], with background levels within soils located in the upper 15 cm at 3.5 mg/kg in the northern part of the county and at 7.0 mg/kg in the southern part. This study acknowledged regionally elevated levels due to larger scale anthropogenic influences.

The objective of the current study was to evaluate the risk to children who play at baseball fields with arsenic at levels above background. The sites studied are located in Miami, Florida U.S. As part of a county wide inspection and sampling effort, both parks were closed in 2014 due to levels of arsenic above the Florida SCTLs. Initial results showed that areas outside the baseball fields showed lower levels of soil contamination and for this reason this assessment was limited to the baseball field areas. Although exposure for others may have been a concern (e.g., maintenance workers), the focus here was on children and their total exposures through adulthood as they used the fields over the years. The study assumed that remediation did not occur and concentrations remained mostly the same at these sites throughout the exposure duration. Although arsenic exposure is detectable in urine, feces, skin, hair, nails, and lungs, no biomarker monitoring was conducted at the time. The community was interested however in a risk assessment to look at potential exposures based on soil concentration levels. Typical risk calculations involve either choosing one concentration value or a range of values that represent various exposure scenarios. These results will then drive a remediation decision. In this study, we evaluated whether altering inputs for the risk calculations would lead to a more representative estimate of exposure for children. Arsenic in soils at the baseball field study sites showed distinct distributions between infield, outfield, and areas adjacent to the fields. Therefore, the study considered various locations on the baseball field and the effects of varying exposure scenarios, including baseball play positions. With a focus on the top layers of soil on the baseball fields, this risk assessment evaluated non-cancer and cancer risks and examined oral, dermal, and inhalation exposure to arsenic.

## 2. Methods

A risk assessment framework was used [31,32] to estimate the risks to children playing on the targeted baseball fields. The approach included the identification of chemical(s) of concern and their concentration levels and distribution (i.e., hazard identification). For the chemical of concern, arsenic, the amount absorbed by a hypothetical person was computed based on various scenarios and routes of exposures (i.e., exposure and dose assessment). This was then followed by an assessment of risk based on toxicological dose response guidelines for the chemical(s) (i.e., risk characterization). Risk was considered separately for adults and children. Lifetime cancer risks are based upon an “age adjusted” basis. This means that we assume the receptor spends various durations being exposed as children with child-like exposure activity and the rest exposed as adults with adult-like exposure activity. Oral, dermal, and inhalation exposures were calculated based on mechanistic equations and required exposure and dose parameters available in the literature and were based on best assumptions across age groups [33].

### 2.1. Hazard Identification

Historical use such as agricultural use, or military use can lead unknowingly to human health concerns when land use changes and areas become public-use lands. The baseball fields at two parks, Chapman and Colonial Fields, were identified with elevated contaminant concentrations after an initial screening of all public playgrounds in Miami-Dade County, Florida, USA. This screening was initiated by Miami-Dade County Department of Regulatory and Economics Resources’ Division of Environmental Resources Management (DERM). The screening was part of DERM’s evaluation of all 263 Miami-Dade County Parks for potential contamination. The source of arsenic at the site is unknown but may be related to the importation of contaminated soil during the development of the fields, or use of pesticides or herbicides that contained arsenic. A possible source of the contamination in these particular baseball fields is monosodium methyl arsonate (MSMA), because it was the most widely used arsenical herbicide prior to its phase out in 2009. Lower infield soil concentrations suggest that the arsenic compound was added to grassier areas for lawn treatment. Although, the organic arsenic compounds, such as MSMA, are considered less toxic than the inorganic forms, these methylated organic acids are considered carcinogenic and management practices for their use are heavily emphasized today [11,34]. In addition studies have shown that the organic MSMA compound may degrade to inorganic forms over time [13].

Sampling and analysis reported in this study was carried out between April and September 2014 through Miami-Dade County Department of Environmental Resources Management, either directly by Miami-Dade County (Colonial) or through contract with CEI Engineers and Contractors, Miami Lakes, FL (Chapman). Sampling strategies were somewhat different for each site given the different groups involved and the fact that subsequent sampling strategies depended upon the results from prior samples at the specific fields. All analyses were conducted by certified laboratories employing routine quality assurance and quality control protocols. The initial screening results from Miami-Dade County triggered more in depth sampling at Chapman and Colonial Fields to identify the specific contaminant(s) of concern and their distribution.

Chapman Field was screened for 20 pesticides and 18 polyaromatic hydrocarbons (PAHs). All concentrations were below residential risk based levels as set by Florida [35]. These risk thresholds are based on aggregate exposures (children to adult) to soil within residential settings. All concentrations were also below the minimum detection limits (MDL) which were in the 0.00023 to 0.12 mg/kg for the pesticides and in the 0.013 to 0.087 mg/kg range for the PAHs. Exceptions were observed for benzo(a)pyrene which was below the practical quantification limit (PQL) of 0.037 mg/kg and for 4,4-DDE which was below the PQL of 0.0037 mg/kg. For more details about the specific chemicals analyzed and their PQLs see FDOH 2015 a,b [36,37].

Both Chapman and Colonial Fields were also screened for 15 metals (aluminum, arsenic, antimony, barium, cadmium, chromium, copper, iron, lead, manganese, mercury, nickel, selenium, silver, zinc). Among these metals, all were below risk based levels with the exception of arsenic. The residential risk based level for arsenic in Florida is 2.1 mg/kg, and samples collected during the initial screening tested at values above this level.

**Figure 1:**
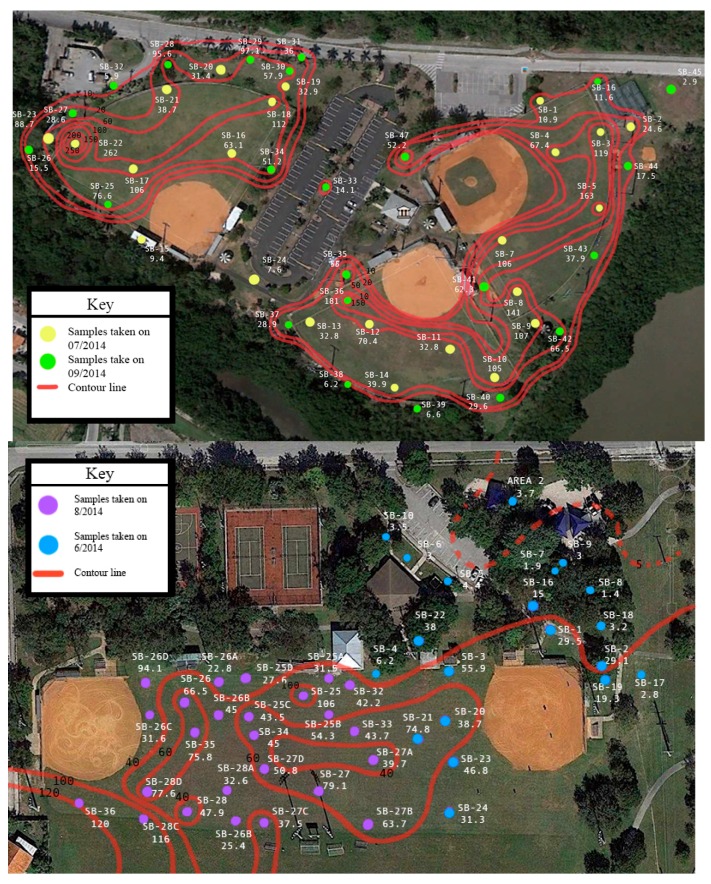
Sampling locations and surface soil arsenic distributions for Chapman (**top**) and Colonial (**bottom**) fields. Top panel formatted from images provided by CEI Engineers and Contractors. Base maps from Google Earth.

Once it was found that arsenic was the only chemical of concern, further soil cores were taken to define concentration distributions. Samples were collected in and around the baseball fields at both parks and at different soil depths. The depth of surface samples typically corresponded to 0–5 or 0–15 cm. Sets of samples at both fields were also collected at depths from 0 to 5 cm and from 5 to 15 cm from the same location. In order to provide a consistent basis of comparison, an equivalent concentration corresponding to 0–15 cm was computed from these sets of cores depending upon the comparisons that are made. Results from the soil samples showed that concentrations in the surface soils were higher than the subsurface soils (Table 1) (*p* < 0.001, students *t*-test, α = 0.05). With respect to sample locations, samples were collected at Chapman Field from the baseball infield, baseball outfield, and in areas adjacent to but outside of the ballfields. At Colonial Field samples were also collected from areas adjacent to the ballfields. However, within the ballfields, samples at Colonial Field were collected only from the outfield. Results show that infield samples were lowest; areas adjacent to the ballfields were at intermediary values; and outfield samples were the highest (Table 2) (*p* < 0.001). The graphical distribution of arsenic concentrations (Figure 1) further illustrates the distinct pattern of arsenic concentrations, showing that arsenic concentrations were highest at both fields in the outfield. Lower levels were observed along the perimeter of the fields with the lowest levels in areas removed from the fields. The infield at Chapman field was characterized by distinctly low concentrations. This distribution in arsenic concentrations suggests differences in arsenic exposure depending upon position that a child may play on the field.

Given the variability in arsenic concentrations at the site, a sensitivity analysis was conducted to evaluate how different arsenic concentrations found at the site could impact risk. Six different concentration values, *C_s_* in Equations (1)–(3), were used to compute risk (Table 3). All of the concentrations corresponded to the upper 15 cm composite average with the exception of the maximum value which corresponded to the maximum single concentration observed from the 0 to 5 cm and 5 to 15 cm soil depth samples. The first set of concentration values corresponded to the 95% upper confidence limit (UCL) concentration for Chapman Field and for Colonial Field. These values were obtained using ProUCL software program (U.S. EPA, Washington, DC, USA) which considers the data size, data skewness, and data distribution when computing the upper 95% UCL of the mean. The remaining four values correspond to: (a) the maximum concentration regardless of soil sample depth, (b) the maximum concentration corresponding to 0–15 cm soil depth, (c) the average infield concentrations for Chapman field and, (d) the 95% UCL based upon levels measured in outfield areas. For this study, we used several concentrations to evaluate the potential exposure at several field positions. Traditionally, when remediation of a site is the driver, a maximum, single concentration is used to conservatively address exposure and health risk should that exposure continue. As we looked at alternate exposures we found that 95% upper confidence limits (UCL) considered distributional skewness and in some cases outliers. As a result the 95% UCL may be a more representative of the concentrations for the exposure scenarios as opposed to using the absolute maximum value. The maximum values for each field (262 mg/kg for Chapman and 120 mg/kg for Colonial) were a factor of three to four times larger than the 95% UCL (63.4 mg/kg for Chapman and 47.7 mg/kg for Colonial).

### 2.2. Computations of Exposure and Dose

Point estimate calculations were used to determine health risk for various exposure scenarios as they relate to recreational baseball fields. The scenarios consisted of multiple periods, including a conservative estimate of exposures throughout a lifetime. The lifetime exposure includes a baby who may visit the field, may later play on or visit the same field as a child and later as an adult. Exposure considered exposure factors from birth to adulthood. Exposures were determined if the person only experienced one of those scenarios and if the person experienced all those scenarios. Since infield and outfield soil concentrations were distinctly different, exposure was also computed based upon an assumed player position of infield or outfield by varying concentrations and other applicable exposure factors. Infield positions included first, second, and third base, short stop, catcher and pitcher, while outfield players included left, right and center fielders. To account for the typical way a game is played with a team spending some time in the field and sometime batting, a scenario was also created for half time infield and half time outfield. One additional scenario was considered and it included that of a child with a condition known as pica which results in elevated ingestion rates of sediments. Pica is common in children between the ages of 2 and 3 [38,39].

The Florida residential risk based levels, SCTLs, used to screen soil contaminant concentrations are based upon a child within a residential setting. The soil concentrations in the current study correspond to a baseball field setting. Because of the mismatch between the scenarios used to establish the Florida residential SCTL and the current study, exposure factors were adjusted using readily-available information to account for baseball scenarios.

Exposure is concerned with how much of a contaminant reaches the human contact boundary (i.e., skin, lungs and mouth), while dose addresses the uptake of the contaminant into the human body following exposure. Exposure and dose to arsenic were assessed using EPA standardized equations [31]. Algorithms used for computing exposure and dose through the oral, *D_s_*, dermal, *D_d_*, and inhalation, *D_i_*, routes are given by Equations (1)–(3) below, respectively.

(1) $$D_{s}=\frac{C_{s}\times IR_{s}\times RBA_{s}\times EF\times CF}{BW}$$

(2) $$D_{d}=\frac{C_{s}\times SA\times AF\times ABS\times EV\times EF\times CF}{BW}$$

(3) $$D_{i}=\frac{C_{s}\times \left(\frac{1}{PEF}\right)\times IR_{a}\times RBA_{a}\times ET\times EF}{BW}$$

Exposure factors found in the equations above for each exposure route (oral, dermal, inhalation) are based on those that correspond to physiological factors and contact patterns at the different ages and are predominantly taken from the EPA Exposure Factors Handbook [40], and also chemical properties of the contaminant. Terms are defined as body weight (*BW*, units of kg), soil intake via ingestion (*IR_s_*, mg/day), surface area of the exposed skin (*SA*, cm^2^), skin adherence factor (*AF*, mg/cm^2^), inhalation rate (*IR_a_*, m^3^/h), and particle emission factor (*PEF*, m^3^/kg),relative bioavailability for ingestion (*RBA_s_*, unitless), skin absorption factor (*ABS*, unitless) and number of sediment loading events per day (*EV*, events/day), relative bioavailability for inhalation, (*RBA_a_*, unitless) and the number of hours of exposure per day (*ET*, h/day). *CF* is a conversion factor. In both Equations (1) and (2), *CF* is equal to 10^−6^ to convert from mg to kg. Exposure duration (*ED*, years), averaging time (*AT*, days), and frequency of exposure (*F*, days/year) were used to compute the exposure factor (*EF*) (Equation (4)) and are based on the duration and time spent at the baseball fields, and opportunities for contact.

(4) $$EF=\frac{F\times ED}{AT}$$

For non-cancer risks *ED* was estimated over the same period as *AT* such that *EF* = *F*. For cancer risks, *ED* is based on time spent at the baseball field for that age group. Toxicity values for cancer were based upon lifetime average daily doses (LADDS) and therefore, *AT*, was assumed over an average life time of 78 years [40] which is then converted to days for input into Equation (4). LADDS were also computed by adding the exposure values across the age groups for cumulative cancer risks [41,42].

For Equations (1)–(3), the values used can be found in Table 4. Pica ingestion rates were applied to the 2 to 3 years age group as this is the age group most prone to pica behavior [38]. A relative bioavailability value of 0.33 was used. This value is based upon a non-human primate model [43] and is consistent with the 2010 U.S. EPA study [44] which found *RBA* values from 10% to 60%. Skin surface areas correspond to the head, hands, arms, and legs. The surface area of the feet was also considered for adolescents less than 21 years of age. A factor of 50% was applied to the surface area values to account for areas covered by clothing or area that did not come in contact with soil. A value of 23 mg/cm^2^, for children playing in mud, was used for the infield players whereas a more conservative value of 0.70, for children’s laying in sediment, was used for outfield players where more grass was found ([40], Table 7-4, the fourth table in Chapter 7). The skin absorption factor for arsenic was set to 0.001 which is the value recommended for inorganics [45]. The number of sediment loading events per day, corresponds to the number of times per day that the soil is removed from the skin and reapplied. This would require one cycle of exposure to the contaminated soil and washing the exposed skin surface area. For the children engaged in a game, a conservative value of 2 was used assuming that at most children will clean-off once per day of exposure. This value is considered conservative because children remain on the field during their games. Siblings of children may be cleaned once per game also allowing for the possibility of 2 events.

For the inhalation route, we used the recommended values for male engaged in short term moderate intensity levels ([46], Table 7-21). Averages across age groups were matched to the 2 to 6 and 6 to 21 years exposure scenarios. Adults 21 years and over were assigned breathing rates of 1.2 m^3^/h according to adult male light activity breathing rates ([40], Table 6-26). Players were assigned values from for male’s short term high activity inhalation rates according to age group ([46], Table 7-21). Relative absorption through inhalation was assumed to be 1. The hours of exposure per day was assumed at 2, which is the assumed time of a game or practice. The soil-to-air particulate emission factor, *PEF*, corresponds to the amount of sediment in air [47].

The *PEF* relies on many factors (Table 5) including the amount of vegetative cover and soil disturbance by wind as seen in Equation (5) [35,48]. For the child through adult scenarios the *PEF* was kept at its original value based on vegetative cover of 50%. However, for the scenarios that considered player position, the *PEF* was adjusted to reflect the vegetative cover for the field area that corresponded to that position. The outfield of a baseball field is typically all grass depending on maintenance and quality of grass. Site visits indicated that these fields were not artificial turf, sometimes found on professional fields and in fact had patches of exposed soil throughout. The infield of only one of the baseball fields was partly covered with grass, but the grass was not around the bases, which is where most of the first, second, and third basemen would spend their time. The *PEF* was adjusted based on 5% vegetative cover for the infield and 85% vegetative cover for the outfield. The *PEF* value as expressed in Equation (5) does not consider disturbance of soil due to players sliding, ball impacts, nor rakes which are sometimes used to groom the infield.

(5) $$PEF=\frac{Q}{C}\times \frac{3600}{0.036\times \left(1-V\right)\times {\left(\frac{U_{m}}{U_{t}}\right)}^{3}\times F\left(x\right)}$$

The exposure factor (Equation (4)) needed to compute exposure and dose (Equations (1)–(3)) is dependent upon the frequency of exposure, *F*, which is the number of days per year an individual could expect to be exposed to the contaminant at the site. In order to estimate the frequency of exposure, two individuals were interviewed between July and August 2014, a park manager and leadership from the baseball-softball association. According to these interviews, Chapman Field Park traditionally hosts two playing seasons per year, a 3-month fall season corresponding to September through November, and a 5-month spring season corresponding to January through May. Teams play typically once per week during the fall season and twice a week during the spring season. Assuming, conservatively, that each team holds two practices prior to every game, a value of 169 days per year was computed for *F*. Less information on play seasons and activities were available for Colonial Field, therefore the same play periods as for Chapman Field were assumed in the exposure calculations. It is possible that more intense or committed players had extended practice and batting practices that might have increased their exposure durations.

### 2.3. Risk Characterization

Once the doses were computed, non-cancer and cancer risks were evaluated. For non-cancer risk, a hazard quotient was computed; this is the ratio between the computed dose and the minimum risk levels (MRLs). A hazard quotient greater than 1 indicates a potential acute or chronic non-cancer risk [50]. For arsenic MRLs were available only for oral routes of exposure. MRLs were not available for dermal nor inhalation routes of exposure. For oral routes of exposure, ATSDR [51] established a minimum risk level (MRL) dose of 5 × 10^−3^ mg/(kg·day) for acute (≤14 days) arsenic effects and a value of 3 × 10^−4^ mg/(kg·day) for chronic effects from arsenic (≥1 year). ATSDR based this MRL on a study of people who drank well water containing inorganic arsenic for many years. This study identified a no observable adverse health effect level (NOAEL) at a dose of 8 × 10^−4^ mg/(kg·day). The NOAEL is the highest dose where no adverse health effects are noted, so doses below this value would not be expected to lead to adverse health effects. At a dose of 1.4 × 10^−2^ mg/(kg·day), the study identified a pattern of skin changes. ATSDR derived their MRL by dividing the NOAEL by an uncertainty factor of 3 for human variability [6].

For cancer, the increased theoretical risk, *R*, was derived by multiplying the estimated dose by the U.S. EPA cancer potency slope factor, *PF*, which for arsenic is 1.500 kg·day/mg for oral ingestion routes, 1.579 kg·day/mg for the dermal route, and 15.050 kg·day/mg for inhalation routes. The potency factor provides a conservative estimate (95% confidence limit) for increased cancer risk. Risk was computed for each route separately and then summed to obtain aggregate risks over all routes of exposures.

Results from risk computations can be put into perspective, with values that can vary from extremely low increased risk (10^−6^) to very high increased risk (10^−1^) (Table 6) [36].

Usually cancer risk is estimated over a lifetime (78 years) but it can also be estimated over a significant portion of the lifetime, 33 years for residential scenarios and 25 years for worker scenarios [36]. In this study cancer risks were computed over a 78-year lifetime but aggregated over different exposure periods like 0.5 to 1 year, 1 to 2 years, 2 to 3 years, 3 to 6 years, 6 to 11 years, 11 to 21 years, 21 to 65 years and 65 to 78 years to account for significant changes in size and other factors like intake rate, body weight, etc. that influence exposure as children age.

## 3. Results

Results include the average daily dose relevant for non-cancer acute and chronic outcomes, and LADDs relevant to cancer outcomes. An emphasis was placed on evaluating age differences, routes of exposure, changing exposure concentrations, pica behavior, and positions of players on the baseball field. Both non-cancer and cancer risks were subsequently computed.

### 3.1. Dose: Age Group Differences and Exposure Routes

Route-specific and aggregate daily dose (Table 7) across age groups for the two baseball fields using the 95th UCL concentrations showed aggregate doses that ranged between 2 to 3 orders of magnitude. Because of the higher concentration found at Chapman (63.4 mg/kg) relative to Colonial (47.7 mg/kg), higher daily doses were found at that field, given that exposure parameters remained the same. The highest aggregate daily dose was found for children aged 0.5 to 1 year, 1 to 2 years, 2 to 6 years, and 6 to 11 years, of 1.1 × 10^−4^, 1.7 × 10^−4^, 1.1 × 10^−4^, and 6.2 × 10^−5^ mg/(kg·day), respectively for Chapman Field. Ingestion dose (first results column in Table 7) contributed from 93.5 percent (for 21 to 65 years age group) to 99.0 percent (for 1 to 2 years age group) to the total aggregate exposure across all age groups for both baseball fields. Dermal dose represented a slightly higher percent of the aggregate dose for adults because ingestion rates drop for this group. Regardless, the ingestion rate was an influential activity factor in overall aggregate doses. The 1 to 2 years group was the highest exposure group, with a higher daily ingestion rate of 200 mg compared to the younger age group whose ingestion rate was 100 mg per day. The 1 to 2 years group also had a higher daily ingestion dose over the 2 to 6 years group because of their lower bodyweight compared to the older age group where both share the same ingestion rate.

LADDs, used to assess cancer risks, were also computed for individual age categories for the cumulative lifetime exposure scenario (Table 8). The relative distribution among exposure routes were similar to those shown in Table 7 above. The LADDs computed for individual categories were 1 to 3 orders of magnitude smaller, as the doses were averaged over a lifetime of 78 years as opposed to averaging over the exposure period. As observed for the non-cancer average daily doses, the cancer LADDs were highest for Chapman Field due to the higher soil arsenic concentrations. Slightly higher LADDs were found for children aged 2 to 6 years and 21 to 65 years of 5.72 × 10^−6^ and 6.84 × 10^−6^ mg/kg, respectively for Chapman. The LADDs seen for the 21 to 65 years group were relatively high given their longer exposure years, whereas aggregate cancer doses for the 2 to 6 years group were influenced by their higher ingestion rates and higher exposure period compared to the 1 to 2 years group with similar ingestion rates. The ingestion route cancer doses were 2 to 3 order of magnitude greater than the dermal doses across age groups and 4 to 5 orders of magnitude greater than the inhalation dose across age groups (e.g., ingestion dose was close to 98% of aggregate dose for the Colonial Field for the 0.5 to 1 year group). A cumulative or a LADD calculated by summing LADDs for each age group can be also found in Table 8, and considered the scenario where an individual experienced arsenic exposures for a lifetime from infant to adult. This person would have higher doses and will have been at increased risks.

### 3.2. Dose: Concentration Scenarios and Pica Behavior

Here we compared the influence of soil exposures concentration on the aggregate daily dose using the highest single concentration found at any field regardless of sample depth (262 mg/kg which occurred at Chapman Field), the highest 0–15 cm concentration (120 mg/kg at Colonial Field), and the 95th UCL across this composite concentration at each field (63.4 mg/kg at Chapman Field and 47.7 mg/kg at Colonial Field) (Table 9). Using the same exposure and time variables across age groups, the daily doses were proportional to the concentrations. As a result the highest concentration at Chapman had some of the higher daily doses ranging from the highest of 7.1 × 10^−4^ mg/(kg·day) for the 1 to 2 year group to 5.4 × 10^−5^ mg/(kg·day) for the 21 to 65 years group. The daily dose, using concentration of 262 mg/kg versus using the 95th UCL composite concentrations of 48 mg/kg for Colonial field produced order-of-magnitude differences in aggregate daily outcomes. As an example, the aggregate daily dose varied from 1.3 × 10^−4^ to 7.1 × 10^−4^ for the 1 to 2 years group, a 313% increase, illustrating the variability of estimating exposures over variable concentrations (Table 9). Aggregate LADDs for the various concentrations relevant for varying concentrations also produced similar percentage differences.

To further evaluate aggregate daily doses, we also considered a child that practiced pica activity during the 2 to 3 years age period using the highest concentration of 262 mg/kg found at Chapman Field (last column of Table 9). This can be considered an overall worst-case exposure scenario that might affect that age category. The ingestion rate was increased from 200 mg to 1000 mg for that age group [52], resulting in a daily aggregate exposure of 2.9 × 10^−3^, the highest aggregate daily dose seen in any exposure scenario, for any age group. For the pica scenario, exposure for age group 3 to 6 years was reduced to non-pica rates as explained by variable changes in Table 4. Although, we have considered an extreme behavior here and its influence on aggregate daily doses, other extreme behaviors are possible, such as a child that rolls routinely in the soil and wears limited clothing exposing increased skin surface areas.

Cumulative lifetime aggregate cancer doses (mg/(kg·day)) were also determined across all age groups, infant to 78 years of age by adding arsenic doses within each group and then aggregating across all groups and all exposure routes (last row in Table 9). This allowed for a scenario of someone that spends time across all groups at the baseball field, first as an infant and young child attending a sibling baseball game, later engaged in the game, and then later as an adult (parent and grandparent) that attended the games with families. The highest concentration at Chapman field of 262 mg/kg produced the highest lifetime aggregate cancer dose of 2.32 × 10^−5^ compared to aggregate cancer doses using the highest composite concentration and 95th UCL concentration found at both fields. All lifetime aggregate cancer doses were heavily influenced by the higher cancer dose for adults aged 21 to 65+ years and not significantly by the exposures at younger ages, even in the case of the pica child (Table 9).

### 3.3. Dose: Consideration of Player Position

In the baseball fields studied, the concentration distributions in the field led to varying exposures and daily doses for the different positions played on a field. Field positions may also affect other exposure factors such as the *PEF* factor and skin adherence based on environmental differences and player behaviors. For concentration, there was an observed difference in the outfield and infield arsenic soil concentrations for Chapman Field. Only outfield concentrations were measured for Colonial Fields and these concentrations were slightly lower than for Chapman Field. As a result, this section focused on utilizing the infield versus outfield concentrations measured at Chapman Field given the availability of infield measurements and the representativeness of the outfield data which was similar to that observed for Colonial Field. Given the availability of data (*n* = 21), the 95th UCL was computed for the outfield concentrations at Chapman Field (87.3 mg/kg). Only four data points were available for the infield concentrations and so the average value (4.8 mg/kg) was used for the infield scenario.

For age group 6 to 11 years, Chapman had an aggregate outfield daily dose of 5.9 × 10^−5^ mg/(kg·day). For these outfield play position scenarios, the younger age group, 6 to 11 years, had the highest daily ingestion, dermal, inhalation and aggregate dose due mostly to lower relative body weights, despite slightly higher inhalation rates for the older group.

The infield ingestion, inhalation and aggregate doses were lower for players of both ages, versus outfield aggregate daily doses due to the lower infield concentrations compared to outfield concentrations, and the fact that ingestion daily doses played the most significant role in aggregate estimates. For dermal exposures across the age groups for infield versus outfield concentrations, variations were observed as influenced by activity patterns and other factors. For example, the infield dermal daily dose for the 6 to 11 years group was 1.0 × 10^−5^ mg/(kg·day), whereas the outfield dermal daily dose for the same age group was lower at 3.8 × 10^−6^ mg/(kg·day). This higher dermal dose was also found for infield players for the older age group. Soil adherence values, relevant for dermal route, were greater at 23 mg/cm^2^ versus 0.7 mg/cm^2^ for infield versus outfield. Adherence has a significant impact despite the higher concentrations in the outfield. The difference between infield and outfield daily doses for inhalation were not as large as expected, because *PEF* values relevant for the inhalation route were higher for infield players, but the higher concentration values in the outfield had more significance in the daily estimates. Infield inhalation daily dose for the older group versus the younger group doses were slightly higher because the older age groups had a higher inhalation rate (Table 10), despite their higher relative body weights.

The players that spend half their time in the infield and half in the outfield experience aggregate daily doses midway between a full-time infield player and a full-time outfielders (numbers are not listed in Table 10 for brevity). We expect that even when players are sitting in the dugout waiting to bat or field, they are still exposed through all three routes to varying degrees. However, it is possible that in the dugout their inhalation rates may go down because they are not running around (i.e., lower exertion and breathing rates), while dermal pressures that influence dermal uptake may not be occurring. The soil may still be adhered to the skin or deposit to the skin. Ingestion exposures may decrease because sweating, wiping brows and cross-transfer of contaminant from fingers to in and around the mouth is lessened while waiting to play. In contrast, players, while bored, might be touching hands to mouth or eating and drinking which facilitates the transfer of soil to the mouth. Until activity patterns are observed and quantified more precisely, their influence on exposures cannot be more accurately determined.

For LADDs (Table 11), aggregate cancer doses were 5.6 × 10^−6^ and 5.5 × 10^−6^ mg/(kg·day), respectively for the 6 to 11 years and the 11 to 21 years age groups at Chapman Field in the outfield. The older age group had only a slightly higher cancer dose for inhalation, largely due to the longer exposure periods found in the older age group (10 years versus 5 years), despite lower body weights for the younger age groups. Older age groups also had higher breathing rates that influenced inhalation exposure. The younger group however had higher ingestion doses, despite shorter exposure periods, due to higher ingestion rates. The cancer doses for infield players were lower (one or two orders of magnitude difference) for players of both ages due to the lower infield concentrations. Although soil adherence values and *PEF* values were higher for infield players for dermal and inhalation routes, the dominant ingestion route and higher concentration values in the outfield had more significance in the cancer calculations. Again, the players that spent half their time in the infield and half in the outfield experience aggregate cancer doses higher than a full-time infield player, but lower than full-time outfielders.

### 3.4. Non-Cancer Risk by Age Group, Exposure Concentrations, and Pica Activity

The highest concentration found at Chapman of 262 mg/kg posed the greatest potential risk for chronic outcomes across all age groups (Table 12). However, most concern exists for the youngest age groups for non-cancer chronic outcomes. Age group 1 to 2 years had the highest hazard quotient (HQ) of 2.37, followed by 1.55 and 1.48 for the 2 to 6 years group and the 0.5 to 1 year group, respectively. We see slight increases in risk for non-cancer chronic outcomes also for 1 to 2 years group for the highest composite concentration from the 0–15 cm soil depth for Colonial Field (HQ 1.08). These at-risk groups demonstrate higher ingestion rates, along with relatively lower body weights. For other scenarios, where a child exposed to the highest soil concentration found at the Chapman Field practicing pica with an ingestion rates up to 1000 mg/day, we see a significant increase in the HQ to 9.69. No non-cancer acute risks were determined for any concentration or age group.

### 3.5. Non-Cancer Risk by Player Position

There does not appear to be any concern for health risks outcomes for players (i.e., HQ < 1) based on infield and outfield concentrations, although some parameters were adjusted for increased dermal and inhalation exposures (Table 13). Only if we assume players exhibit higher ingestion rates, for example by wiping their lips while sweating during play, would exposures be of concern for chronic, acute or cancer outcomes, given that ingestion rates heavily influence aggregate exposures. Other factors that influenced inhalation and dermal exposure should still also be explored such as increased particle emission factors due to soil disturbance and greater soil adherence like sliding in mud, and exposures of the back or torso. It is possible that some players spent greater times per day and for certain weeks at these baseball fields. These players may have participated in more than one team, or have engaged in additional training and batting practices.

### 3.6. Cancer Risk by Age Group, Exposure Concentrations, and Pica-Activity

For any single age group and for the arsenic concentrations modeled, most increased risks were “very low” or “extremely low” increased risks (at or greater than 10^−5^, 10^−6^, respectively) (Table 14). As expected, the cancer risk estimates using the highest concentration found at Chapman of 262 mg/m^3^ resulted in the higher cancer risks for child age group 2 to 6 years and adult age group 21 to 65 years, over other concentrations modeled. For the younger group, relatively higher ingestion rates, along with relatively lower body weights, played a role. For the older age, the longer years of exposure (i.e., 44 years) contribute to the increased risk. The child that practiced pica activity and was exposed to the highest concentration at Chapman, could have experienced a larger incremental risk to cancer during this year of exposure over their lifetime. A cumulative or lifetime aggregate cancer risk (i.e., combining exposures across the age groups) was determined for these scenarios, where a Chapman concentration of 262 mg/kg with and without a child practicing pica activity, lead to “low” increased cancer risks of 2.7 × 10^−4^ and 1.6 × 10^−4^, respectively.

### 3.7. Cancer Risk by Player Position

Cancer health risk outcomes for players were “extremely low” for highest exposed groups, which were all outfield players at Chapman field (Table 15). The lower risks for the infield group were due to lower infield concentrations. The difference in exposures and risks widened between infield and outfield players when cancer risks were accumulated over the age groups.

## 4. Discussion

The main objective of this research project was to determine whether the elevated levels of arsenic in soils found across two baseball fields posed a significant increase in health risk to players and their families that may have used the fields over the years. SCTLs are typically derived from residential exposures which consist of distinctly different behaviors and exposures than those on a baseball field. This study conducted a relevant risk assessment to evaluate non-cancer acute and chronic and cancer outcomes specific to baseball field exposures, given data that was readily available.

For non-cancer chronic outcomes, the major findings were the following: (1) when the highest soil concentrations were used in the exposure estimates, concerning risks were computed for the three age groups from 0.5 to 6 years for Chapman Field and for the age group 1–2 years for Colonial Field (HQ between 1 and 3), and when (2) the highest concentration at Chapman was combined in the exposure estimate with a child 2 to 3 year of age that practices pica-activity (i.e., ingestion rate of 1000 mg/day), the HQ risk increased to over 9. Although chronic non-cancer outcomes were estimated, no non-cancer acute outcomes were of concern for any single age group or exposure scenario. In addition, no non-cancer risks, acute and chronic, were determined for player positions (age groups 6 to 21 years), despite some changes in parameters that increased the exposures. The significantly higher concentrations in the outfield field meant outfield players experienced higher exposures, despite more access and contact with soils in the infield.

For cancer probabilities across age groups and exposure scenarios, the risks ranged from “extremely low” (near 1 in a million chance) to “low” (near 1 in ten thousand) increased risk. These risks fit within U.S. EPA’s target risk range for cancer which is 1 in million chance (10^−6^) to 1 in ten thousand (10^−4^). Pica ingestion, soil concentrations, and duration increased the probability of cancer risk as the respective exposure factor increased to its maximum. For instance, pica ingestion had extreme intake rates or lifetime exposure utilized the longest duration. The scenarios where one of the exposure factors was maxed typically estimated the highest cancer risk. In evaluating less than lifetime exposures, cancer risks in some scenarios estimated “low” (near 1 in ten thousand chance) cancer risks, when an individual experienced continued exposures on the fields. There are a few considerations in scrutinizing these cancer risk estimates. Although estimates assume continuous exposure for these age categories, there may be gaps in lifetime exposures that occur for these baseball exposure scenarios. For example, exposure to arsenic on these fields may not be occurring during (1) the period when an individual turns 21 and the time when they have a child that plays baseball and (2) the period when a parent has a child that stops playing baseball to when they have a grandchild that plays baseball. Other exposure gaps may occur in this population for those who do not have children or grandchildren, and only play baseball as children. These time gaps periods are especially important to consider when aggregating these risks over all age groups for an individual. One of the fields, Chapman Fields, was constructed in 1972 and closed in 2014 (open 42 years), which may also limit the actual years of usage and exposure as modeled across the age groups for cancer outcomes.

Ingestion exposures was found to be particularly significant in the calculations, where regardless of age group or exposure scenario, it contributed over 90% to aggregate doses in the estimate of non-cancer and cancer risks estimates. This was reflected in the increased risks for children with pica-activity due to higher ingestion rates. In general, only under more conservative estimates (using highest concentrations or looking at extreme behaviors) were exposure and health risks determined to be undesirable for particular age groups using the fields. The highest concentration may overestimate realistic and continued exposures over time. When the 95% upper confidence levels for the soil concentration across the fields were used to compute risk, the increased risk resulted in non-concerning risks levels for non-cancer and cancer outcomes.

Arsenic exposures are not uncommon in the United States, and it is recognized that exposures through food and water can occur. Population exposure to inorganic arsenic largely occurs through drinking water and consumption of rice. Recent modeling predictions, using the probabilistic model Stochastic Human Exposure and Dose Simulation (SHEDS) model for children 1 to 2 years of age estimated bioaccessible gastrointestinal raw rice exposures were 0.5 µg/day (95th percentile is 2.5 µg/day) [53]. Exposures for arsenic from drinking water for children 1 to 2 years of age, across the U.S. population, was estimated at 1.2 µg/day (95th percentile is 3.7 µg/day), for total water intake. The model incorporated sampling data from drinking water facilities and rice composites across multiple states, along with population consumption patterns and in vitro extraction procedures for gastrointestinal bio-accessible data [53]. The highest rice and water consumption rates and resulting exposures occurred for Tribal, Asian and Pacific, and other Hispanic subpopulations. In addition, all children aged 1–2 years (except non-hispanic white) were at greater risk based on their consumption patterns and lower bodyweights.

The necessity for action in a community to reduce exposures, must consider all sources of exposures. For the age group of 1 to 2 years in this study and using the highest soil level found, the total daily aggregate dose was found to be 0.71 µg/kg·day. Using the 95th percentile for arsenic rice exposures of 2.5 µg/day and 95th percentile for water intake of 3.7 µg/day from the SHEDS study [53], exposure increased for a hypothetical child on this baseball field by 10.3%. All other sources of arsenic in the environment for this hypothetical child had not been estimated. Exposure to arsenic may occur from other school and home sources and from inhalation in the environment from other contaminated soils. When considering acute, chronic and cancer outcomes, exposure to other metals/metalloids for additive or synergistic effects should also be considered, along with the ability to best control sources.

Other studies have been conducted to evaluate children total exposures in a community. In a study to quantify children (aged 5–8 years, *n* = 70 subjects) exposures to arsenic in an urban environment of China the median and 75 percentile values were reported. The median non-carcinogenic hazard quotients were 4.61, 0.35, and 0.00062 (5.32, 0.38, and 0.00068 for the 75th percentile) for the ingestion, dermal and inhalation routes, respectively. These values were based on school, home and indoor and outdoor exposures, indicating unacceptable risks for the ingestion route [54]. Risks were estimated using a similar exposure and risk approach, based upon soil, dust, drinking water and food media exposures. Median concentrations of arsenic found in this urban industrialized environment were reported as 44.2 mg/kg, 75.8 mg/kg, 0.38 µg/m^3^, 0.11 µg/L, and 0.07 mg/kg for soil, dust, PM_10_, drinking water, and duplicate food samples. The 75th percentile concentrations were 84.0 mg/kg, 156.8 mg/kg, 0.48 µg/m^3^, 0.11 µg/L, and 0.26 mg/kg for these same matrices, respectively [54]. The majority of arsenic was suspected to come from battery mining and battery manufacturing in this area of China (i.e., industrial city in Hunan Province). Carcinogenic risks were also found to be high for arsenic from food and dust ingestion. In this study, they found relatively high environmental concentrations throughout the community, which drove total exposure, influenced by an industrial settings and their more widespread use of arsenic. For the baseball fields in this study, exposure times were limited to 2 h per day and for about half the year, but soil arsenic concentrations were higher (compared to the Cao et al. [54] study) in some areas of these baseball fields. For some age groups and for some behaviors (pica activity) we found comparable HQ’s for just these 2 h of play at the baseball fields. The Cao study [54] did consider exposure to other metals in the environment in light of cumulative health risks whereas we evaluated pica behavior which provided similar elevated HQ values.

There are a number of limitations or considerations in this risk analysis. In particular, research regarding baseball field behavior is lacking. Direct observations by videotaping [16,55,56,57] or notetaking can help provide information on player and family behavior at the field. This information can be used to refine the estimates for contact activities with soil (e.g., sliding, ingestion rates). Given the nature of play activities in wet and dry soil, and under contact scenarios, adherence studies such as hand presses can be performed to improve mass soil adherence to skin estimates [58,59]. Pre-recorded baseball game footage can be used to gather some of this relevant information. Macro-surveys can help improve the estimates of time spent on fields, hygiene activities that influence exposures, and other more longitudinal family engagement with the sport. Observational strategies can also be used to better understand proximity effects based on a player’s position and relevance to field concentration [60]. Thiessen polygons are a simple proposed method to determine the arsenic concentrations associated with various positions during field play. Thiessen polygons are polygons with areas defined by boundaries created by the perpendicular bisectors of lines between the sampling points [61]. These polygons can be used to separate the baseball field area into distinct regions with characteristic soil concentrations. An alternate approach would be to consider the gradual variation (or contouring) of the concentrations as shown in Figure 1. In either case child location and time spent at that location can be related to the concentration. Depending upon the child’s play activities (time and location) more representative concentrations can be identified for the child’s exposure as opposed to using the maximum values (extreme maximum or 95% upper confidence limit). There are other aspects that can be considered to improve estimates. During a baseball game, for example, disturbance of the field by players is expected and this disturbance can potentially increase the *PEF* factor and the player inhalation exposure. Further calculations can consider refinement of the *PEF* based on disturbance. More accurate measurements for PEF are obtainable by taking field measurements using particle samplers during play activities. Here we considered soil that adheres to skin based on soil adherence measures used in previous studies in various play and soil environments. Skin adherence of metals (e.g., arsenic) to the skin via deposition under haze conditions was determined using wipe samples by Cao et al. [17] for various populations including children. This study theorized that deposition occurred as a mix of atmospheric particles and ground dust. The study found that soil mass totals found on each person’s skin was influenced by haze level, gender and behavior patterns [17]. To analyze deposition to the skin based on play activities that occur on baseball fields, a soil adherence study can be implemented where wipe samples can be taken on exposed areas of the body following 2 h of play for a number of subjects.

Not all risks have been captured here in these assessments. For example, an extreme peak exposure may result in a missed acute effect for a day or a few days (i.e., high ingestion rate on one day and not averaged over the year of exposure). In addition, abrasions to the skin can increase the absorption rate through skin for arsenic, or existing health problems can increase risks even at low exposure levels, not considered in MRLs and slope factors for health risks. There is additional uncertainty in the time-periods of exposure, accumulating dose and their relationships to MRLs, and cancer potency factors in deciding definitive risk. Arsenic’s acute MRL was derived for 14 days or less of exposure, while non-cancer chronic MRLs were derived for exposure a year or greater. No intermediate MRL has been derived for arsenic [6]. The oral MRLs have been applied in this analysis to the aggregate doses for a determination of risk; however health outcomes may vary based on routes and actual durations of exposure. In practice, constant slope factors are utilized for arsenic risks. However, some studies argue for discontinuities in the cancer slope factor [62]. In addition, still much remains to be understood about the uptake rates, and the metabolic and toxicity pathways for arsenic, where some research has shown variation across individuals (genes and age dependency may play a role), and across the chemical forms of arsenic [63].

## 5. Conclusions

A risk assessment was conducted in this study to evaluate non-cancer acute and chronic and cancer outcomes specific to baseball field exposures, given data that was readily available. Concerning non-cancer chronic exposures, risks were determined only in cases where the highest soil concentrations were used in the estimates for the very young and for children practicing pica activity. In addition, very low to extremely low cancer risks were determined across the age groups and players groups in this study. Limitations exist in this study with respect to lack of precise activity patterns for this population and exposure distributions for arsenic concentrations over time.

In conclusion, children can unknowingly play in areas that are contaminated, and therefore sampling should be encouraged in areas where children frequent (i.e., playgrounds, parks, playfields) to determine soil hazard level, and conduct risk assessments to understand human activities that increase risks. This study is not a comprehensive assessment of total exposure to arsenic for this population, where other environmental sources are likely. Decisions to remediate public places should be based on total exposures for a particular population.

Apart from closing fields and replacing top soil by health departments, there are steps families can make to increases safety such as washing hands more frequently and wearing appropriate clothing in soil covered areas. As a community however, every effort should be made to keep parks and play areas open and safe. Closed parks remove the opportunity for family and community events important for the well-being of all; exercise and game activities are important to children’s health. The two baseball fields were closed once the contamination became known and a regional meeting was held to discuss the health and environmental impacts. The county plans to remediate these two fields by removing or covering the top soil, and authors are in agreement with this precautionary decision.

## Figures and Tables

**Table 1:** Arsenic concentrations with depth at Chapman and Colonial Fields. These results include all samples collected regardless of location (infield, outfield, outside ballfields).

Depth (cm)	Chapman *		Depth (cm)	Colonial	
	mg/kg	*n*		mg/kg	*n*
0–5	58.5	48	0–15	40.7	53
5–15	44.1	47			
15–61	19.7	59	15–61	17.5	26
61–122	15.2	24			
122–152	9.6	24			

* An additional 13 surface samples were collected for the 0–15 cm depth for Chapman. The average of these samples was 29.6 mg/kg. These results are biased low because they were collected early during the sampling study during a time that the location of the contamination was unknown. Once the contamination location was found, sampling then proceeded in the more contaminated areas to identify the distribution with depth.

**Table 2:** Arsenic concentrations by location at Chapman and Colonial Fields. These results include all surface samples collected regardless of depth (surficial, 0–5 cm, 0–15 cm).

Location	Chapman		Colonial	
	mg/kg	*n*	mg/kg	*n*
Infield	4.8	4	N/A	0
Outfield	94.6	21	47.9	40
Adjacent Areas	33.0	36	12.0	13

**Table 3:** Arsenic soil concentrations (*C_s_*) across fields/areas computed for surface soil samples (depth 0–5 cm and 0–15 cm).

Condition Evaluated	Concentrations, mg/kg		Number of Data Points and Distributional Descriptions		Notes
	Chapman	Colonial	Chapman	Colonial	
95% upper confidence limit, all samples (0–15 cm depth)	**63.4**	**47.7**	60 observations, non-parametric distribution	53 observations, normal distribution	Across all concentrations taken in and around fields
Highest (0–5 cm or 0–15 cm)	**262**	120	61 observations	54 observations	Highest values for both fields found in the outfield. The Chapman sample corresponded to a 0–5 cm depth
Highest (0–15 cm depth)	118	**120**	60 observations	53 observations	Combination of direct measures and equivalent composites for 0–15 cm depth
Average (0–15 cm depth)	43.9	40.7	60 observations	53 observations	Combination of direct measures and equivalent composites for 0–15 cm depth. Samples taken within and around fields
Average, infield only (0–15 cm depth)	**4.8**	NA	4	NA *	Three values for Chapman, no infield values for Colonial
Average, outfield only (0–15 cm depth)	76.5	50.0	21 observations	40 observations	NA
95% upper confidence limit, outfield only (0–15 cm depth)	**87.3**	57.7	21 observations, normal distribution	40 observations, normal distribution	Only outfield concentrations

* NA = Not applicable. Bold: The numbers in bold are used in subsequent sections to evaluate the impacts of concentration on dose.

**Table 4:** Age group and player position related exposure factors.

Age Groups	Body Weight, *BW*, (kg)	Soil Intake, *IR_s_*, (mg/day)		Surface Area of the Skin, *SA*, (cm^2^)	Adherence Factor, *AF*, (mg/cm^2^)	Inhalation Rate, *IR_a_*, (m^3^/h)	Particle Emission Factor, *PEF* (m^3^/kg)
		Upper Percentile	Pica Child				
0.5 to 1 year	9.2	100		1450	0.2	0.87	1.24 × 10^9^
1 to 2 years	11.4	200		1705	0.2	1.28	1.24 × 10^9^
2 to 3 years	13.8	NA *	1000	1795	0.2	1.28	1.24 × 10^9^
2 to 6 years	17.4	200		2129	0.2	1.28	1.24 × 10^9^
3 to 6 years	18.6	200		2240	0.2	1.28	1.24 × 10^9^
6 to 11 years	31.8	200		3260	0.2	1.34	1.24 × 10^9^
11 to 21 years	64.2	200		5206	0.2	1.66	1.24 × 10^9^
21 to 65 years	80	100		5690	0.2	1.20	1.24 × 10^9^
65+ years	76	100		5690	0.07	1.20	1.24 × 10^9^
6 to 11 years, infield	31.8	200		3260	23	2.62	6.53 × 10^8^
11 to 21 years, infield	64.2	200		5207	23	3.12	6.53 × 10^8^
6 to 11 years, outfield	31.8	200		3260	0.7	2.62	4.14 × 10^9^
11 to 21 years, outfield	64.2	200		5207	0.7	3.12	4.14 × 10^9^

* NA = Not Applicable.

**Table 5:** Parameters used in the calculation of *PEF*.

*Q*/*C*	85.61	Inverse of a Mean Concentration at Center of a 0.5-Acre Square Source (g/m^2^-s per kg/m^3^) with Undisturbed Soil. Default Value for Miami, FL, USA [47], Where *C* Is an Average Annual Concentration and *Q* Is the Area Source Emmission Rate.
*V*	Variable from 0 to 1	Fraction of vegetative cover. 0.5 used for average conditions, 0.05 used for infield, and 0.85 used for outfield.
*U_m_*	4.69	Mean annual wind speed (m/s). Based on U.S. national data, and is similar to the annual average wind speed measured in Florida [35].
*U_t_*	11.32	Equivalent threshold value of wind speed at 7 m (m/s).
*F*(*x*)	0.194	Function dependent on Um/Ut (unitless) [49].
	0.036	Respirable fraction in g/m^2^-h
	3600	Conversion factor, s/h

**Table 6:** Interpretation of computed risk values.

Value	Risk Level
1 in 10 (10^−1^)	“very high” increased risk
1 in 100 (10^−2^)	“high” increased risk
1 in 1000 (10^−3^)	“moderate” increased risk
1 in 10,000 (10^−4^)	“low” increased risk
1 in 100,000 (10^−5^)	“very low” increased risk
1 in 1,000,000 (10^−6^)	“extremely low” increased risk

**Table 7:** Route and aggregate daily dose, mg/(kg·day), using 95th UCL composite concentrations found at the 0–15 cm soil depth across age groups.

Age Group	Daily Dose, mg/(kg·day)							
	Ingestion		Dermal		Inhalation		Aggregate	
	Chapman	Colonial	Chapman	Colonial	Chapman	Colonial	Chapman	Colonial
0.5 to 1 year	1.1 × 10^−4^	7.9 × 10^−5^	1.9 × 10^−6^	1.4 × 10^−6^	4.5 × 10^−9^	3.4 × 10^−9^	1.1 × 10^−4^	8.1 × 10^−5^
1 to 2 years	1.7 × 10^−4^	1.3 × 10^−5^	1.8 × 10^−6^	1.3 × 10^−6^	5.3 × 10^−9^	4.0 × 10^−9^	1.7 × 10^−4^	1.3 × 10^−4^
2 to 6 years	1.1 × 10^−4^	8.4 × 10^−5^	1.4 × 10^−6^	1.1 × 10^−6^	3.5 × 10^−9^	2.6 × 10^−9^	1.1 × 10^−4^	8.5 × 10^−5^
6 to 11 years	6.1 × 10^−5^	4.6 × 10^−5^	1.2 × 10^−6^	9.1 × 10^−7^	2.0 × 10^−9^	1.5 × 10^−9^	6.2 × 10^−5^	4.7 × 10^−5^
11 to 21 years	3.0 × 10^−5^	2.3 × 10^−5^	9.5 × 10^−7^	7.2 × 10^−7^	1.2 × 10^−9^	9.2 × 10^−10^	3.1 × 10^−5^	2.3 × 10^−5^
21 to 65 years	1.2 × 10^−5^	9.1 × 10^−6^	8.4 × 10^−7^	6.3 × 10^−7^	7.1 × 10^−10^	2.1 × 10^−9^	1.3 × 10^−5^	9.8 × 10^−6^
65+ years	1.3 × 10^−5^	9.6 × 10^−6^	3.1 × 10^−7^	2.3 × 10^−7^	7.5 × 10^−10^	2.3 × 10^−9^	1.3 × 10^−5^	9.8 × 10^−6^

**Table 8:** Route and aggregate LADDs using 95th UCL concentration found at the 0–15 cm soil depth across age groups.

Age Group	LADDs, mg/(kg·day)							
	Ingestion		Dermal		Inhalation		Aggregate	
	Chapman	Colonial	Chapman	Colonial	Chapman	Colonial	Chapman	Colonial
0.5 to 1 year	6.8 × 10^−7^	5.1 × 10^−7^	1.2 × 10^−8^	8.9 × 10^−9^	2.9 × 10^−11^	2.2 × 10^−11^	6.8 × 10^−7^	5.2 × 10^−7^
1 to 2 years	2.2 × 10^−6^	1.6 × 10^−6^	2.3 × 10^−8^	1.7 × 10^−8^	6.9 × 10^−11^	5.2 × 10^−11^	2.2 × 10^−6^	1.7 × 10^−6^
2 to 6 years	5.7 × 10^−6^	4.3 × 10^−6^	7.4 × 10^−8^	5.6 × 10^−8^	1.8 × 10^−10^	1.3 × 10^−10^	5.7 × 10^−6^	4.4 × 10^−6^
6 to 11 years	3.9 × 10^−6^	2.9 × 10^−6^	7.7 × 10^−8^	5.8 × 10^−8^	1.3 × 10^−10^	9.6 × 10^−11^	3.9 × 10^−6^	3.0 × 10^−6^
11 to 21 years	3.9 × 10^−6^	2.9 × 10^−6^	1.2 × 10^−7^	9.2 × 10^−8^	1.6 × 10^−10^	1.2 × 10^−10^	3.9 × 10^−6^	3.0 × 10^−6^
21 to 65 years	6.8 × 10^−6^	5.1 × 10^−6^	4.7 × 10^−7^	3.6 × 10^−7^	4.1 × 10^−10^	1.2 × 10^−10^	6.8 × 10^−6^	5.5 × 10^−6^
65+ years	2.1 × 10^−6^	1.6 × 10^−6^	5.1 × 10^−8^	3.9 × 10^−8^	1.3 × 10^−10^	3.8 × 10^−10^	2.1 × 10^−6^	1.6 × 10^−6^
Cumulative LADDs	2.5 × 10^−5^	1.9 × 10^−5^	8.3 × 10^−7^	6.3 × 10^−7^	1.8 × 10^−8^	1.7 × 10^−8^	2.6 × 10^−5^	2.0 × 10^−5^

**Table 9:** Arsenic daily doses for age groups across exposure concentrations and for pica-behavior.

	Aggregate Daily Dose, mg/(kg·day), Using the:				
Age Group	Highest Single Sample	Highest Composite Concentration Found at the 0–15 cm Soil Depth	95th UCL Composite Concentration Found at the 0–15 cm Soil Depth		Highest Single Sample and Considering Pica Activity for 2 to 3 Years Group
	Chapman	Colonial	Chapman	Colonial	Chapman
	262 mg/kg	120 mg/kg	63.4 mg/kg	47.7 mg/kg	262 mg/kg
0.5 to 1 year	4.4 × 10^−4^	2.0 × 10^−4^	1.1 × 10^−4^	8.1 × 10^−5^	4.4 × 10^−4^
1 to 2 years	7.1 × 10^−4^	3.3 × 10^−4^	1.7 × 10^−4^	1.3 × 10^−4^	7.1 × 10^−4^
2 to 3 years	NA *	NA	NA	NA	2.9 × 10^−3^
3 to 6 years	NA	NA	NA	NA	4.4 × 10^−4^
2 to 6 years	4.7 × 10^−4^	2.1 × 10^−4^	1.1 × 10^−4^	8.5 × 10^−5^	NA
6 to 11 years	2.6 × 10^−4^	1.2 × 10^−4^	6.2 × 10^−5^	4.7 × 10^−5^	2.6 × 10^−4^
11 to 21 years	1.3 × 10^−4^	5.9 × 10^−5^	3.1 × 10^−5^	2.3 × 10^−5^	1.3 × 10^−4^
21 to 65 years	5.4 × 10^−5^	2.5 × 10^−5^	1.3 × 10^−5^	9.8 × 10^−6^	5.4 × 10^−5^
65+ years	5.4 × 10^−5^	2.5 × 10^−5^	1.3 × 10^−5^	9.8 × 10^−6^	5.4 × 10^−5^

* NA = Not applicable.

**Table 10:** Route and aggregate daily dose (mg/(kg·day)) by player position and age, using the 95th UCL outfield concentration and average infield concentrations for Chapman Field.

Age Group	Daily Dose, mg/(kg·day)							
	Ingestion		Dermal		Inhalation		Aggregate	
	Infield	Outfield	Infield	Outfield	Infield	Outfield	Infield	Outfield
6 to 11 years	4.6 × 10^−6^	8.4 × 10^−5^	1.0 × 10^−5^	3.8 × 10^−6^	2.9 × 10^−10^	5.4 × 10^−9^	1.5 × 10^−5^	5.9 × 10^−5^
11 to 21 years	2.3 × 10^−6^	4.2 × 10^−5^	8.2 × 10^−6^	3.0 × 10^−6^	3.3 × 10^−10^	9.5 × 10^−10^	1.1 × 10^−5^	3.1 × 10^−5^

**Table 11:** Route and aggregate cancer dose (mg/kg) by player position and age, using 95th percentile UCL outfield composite concentrations and average infield concentration for Chapman Field.

Age Group	LADDs, mg/(kg·day)							
	Ingestion		Dermal		Inhalation		Aggregate	
	Infield	Outfield	Infield	Outfield	Infield	Outfield	Infield	Outfield
6 to 11 years	1.5 × 10^−7^	5.4 × 10^−6^	6.4 × 10^−9^	1.9 × 10^−7^	1.9 × 10^−11^	3.4 × 10^−10^	1.53 × 10^−7^	5.6 × 10^−6^
11 to 21 years	1.5 × 10^−7^	5.3 × 10^−6^	1.2 × 10^−7^	1.9 × 10^−7^	2.2 × 10^−11^	4.1 × 10^−10^	2.61 × 10^−7^	5.5 × 10^−6^
Cumulative LADD (6 to 21 years)	2.9 × 10^−7^	1.1 × 10^−5^	1.2 × 10^−7^	3.8 × 10^−7^	4.1 × 10^−11^	7.5 × 10^−10^	4.14 × 10^−7^	1.1 × 10^−5^

**Table 12:** Aggregate non-cancer chronic and acute hazard quotients across age groups for concentration scenarios.

	Aggregate Non-Cancer Hazard Quotients Using the:									
Age Group	Highest Single Sample		Highest Composite Concentration Found at the 0–15 cm Soil Depth		95th UCL Composite Concentration Found at the 0–15 cm soil Depth				Highest Single Sample and Considering Pica Activity for 2 to 3 Years Group	
	Chapman		Colonial		Chapman		Colonial		Chapman	
	262 mg/kg		120 mg/kg		63.4 mg/kg		47.7 mg/kg		262 mg/kg	
	Acute	Chronic	Acute	Chronic	Acute	Chronic	Acute	Chronic	Acute	Chronic
0.5 to 1 year	0.09	**1.48**	0.04	0.68	0.02	0.36	0.02	0.27	0.09	1.48
1 to 2 years	0.14	**2.37**	0.06	**1.08**	0.03	0.57	0.03	0.43	0.14	**2.37**
2 to 3 years	NA *	NA	NA	NA	NA	NA	NA	NA	0.58	**9.69**
3 to 6 years	NA	NA	NA	NA	NA	NA	NA	NA	0.09	**1.45**
2 to 6 years	0.09	**1.55**	0.04	0.71	0.02	0.38	0.02	0.28	NA	NA
6 to 11 years	0.05	0.86	0.02	0.39	0.01	0.21	0.01	0.16	0.05	0.86
11 to 21 years	0.03	0.43	0.01	0.20	0.01	0.10	0.00	0.08	0.03	0.43
21 to 65 years	0.01	0.18	0.00	0.08	0.00	0.04	0.00	0.03	0.01	0.18
65+ years	0.01	0.18	0.00	0.08	0.00	0.04	0.00	0.03	0.01	0.18

* NA = Not applicable. Bold: The numbers in bold correspond to hazard quotients greater than one.

**Table 13:** Aggregate non-cancer chronic and acute hazard quotient for player positions using average infield and 95th UCL outfield concentrations at Chapman Field.

Age Group	Acute		Chronic	
	Infield	Outfield	Infield	Outfield
6 to 11 years	0.00	0.02	0.05	0.30
11 to 21 years	0.00	0.01	0.03	0.15

**Table 14:** Aggregate cancer risks across various age groups for varying concentration scenarios, and pica activity.

Age Group	Aggregate Cancer Risks Using the:				
	Highest Single Sample	Highest Composite Concentration Found at the 0–15 cm Soil Depth	Using 95th UCL Composite Concentration Found at the 0–15 cm Soil Depth		Highest Single Sample and Considering Pica Activity for 2 to 3 Years Group
	Chapman	Colonial	Colonial	Chapman	Chapman
	262 mg/kg	120 mg/kg	63.4 mg/kg	47.7 mg/kg	262 mg/kg
0.5 to 1 year	4.3 × 10^−6^	2.0 × 10^−6^	7.8 × 10^−7^	1.0 × 10^−6^	4.3 × 10^−6^
1 to 2 years	1.4 × 10^−5^	6.3 × 10^−6^	2.5 × 10^−6^	3.3 × 10^−6^	1.4 × 10^−5^
2 to 3 years	NA	NA	NA	NA	5.6 × 10^−5^
	NA	NA	NA	NA	2.5 × 10^−5^
2 to 6 years	3.6 × 10^−5^	1.6 × 10^−5^	6.5 × 10^−6^	8.6 × 10^−6^	NA
6 to 11 years	2.5 × 10^−5^	1.1 × 10^−5^	4.5 × 10^−6^	5.9 × 10^−6^	2.5 × 10^−5^
11 to 21 years	2.5 × 10^−5^	1.1 × 10^−5^	4.5 × 10^−6^	5.8 × 10^−6^	2.5 × 10^−5^
21 to 65 years	4.5 × 10^−5^	2.1 × 10^−5^	8.3 × 10^−6^	1.0 × 10^−5^	4.5 × 10^−5^
65+ years	1.4 × 10^−5^	6.2 × 10^−6^	2.5 × 10^−6^	3.2 × 10^−6^	1.4 × 10^−5^
Cumulative Risks	**1.6 × 10^−4^**	7.5 × 10^−5^	3.0 × 10^−5^	4.0 × 10^−5^	**2.7 × 10^−4^**

NA = Not applicable. Bold: Numbers in bold correspond to the highest cumulative risk values.

**Table 15:** Aggregate cancer risks for player positions using average infield concentrations and 95th UCL outfield concentrations at Chapman Field.

Age Group	Infield	Outfield
6 to 11 years	2.3 × 10^−7^	8.4 × 10^−6^
11 to 21 years	4.0 × 10^−7^	8.3 × 10^−6^
Cumulative Risks (6 to 21 years)	6.3 × 10^−7^	1.7 × 10^−5^
